# Study on the expression of p53, PTEN, and p16 in endometrial polyps of breast cancer patients receiving endocrine therapy: a comparative study of immunohistochemistry

**DOI:** 10.3389/fonc.2026.1848585

**Published:** 2026-08-07

**Authors:** Yiwei Du, Jianting Ma

**Affiliations:** Department of Obstetrics and Gynecology, The Affiliated Yangming Hospital of Ningbo University, Ningbo, Zhejiang, China

**Keywords:** breast cancer, endocrine therapy, endometrial polyps, immune expression, immunohistochemistry

## Abstract

**Objective:**

This study aims to compare the expression of p53, PTEN, and p16 among three groups: endometrial polyps of breast cancer patients receiving endocrine therapy, common sporadic endometrial polyps, and endometrial carcinoma (type I). This study also analyzes the correlation between the immunohistochemical expression of these markers and the clinical characteristics of breast cancer patients receiving endocrine therapy.

**Methods:**

From 1 December 2022, to 31 December 2023, we retrospectively enrolled 93 breast cancer patients receiving endocrine therapy with endometrial polyps, 60 patients with common sporadic endometrial polyps, and 48 patients with endometrial carcinoma (type I) (all cases were retrieved from our hospital). The expression of p53, PTEN, and p16 was detected using immunohistochemistry. Spearman’s correlation analysis was conducted to explore factors associated with p16 and PTEN expression in the endocrine therapy cohort.

**Results:**

No statistically significant difference in the p53 expression distribution was observed among the tamoxifen (TAM), toremifene (TOR), endometrial polyps (EP), and endometrial carcinoma (EC) groups regardless of menopausal status. In premenopausal patients, the semi-quantitative immunohistochemical (IHC) scores of p16 and PTEN in the TAM and TOR groups were intermediate between those of EP and EC groups (Holm-adjusted *P* < 0.05). This specific pattern was not observed in the postmenopausal group. In the endocrine therapy-treated cohort, Spearman’s correlation analysis revealed that the semi-quantitative IHC score of p16 was positively correlated with age, blood lipids, and BMI; negatively correlated with abnormal uterine bleeding and endometrial thickness (*P* < 0.05); the semi-quantitative IHC score of PTEN was negatively correlated with menopausal status and diabetes mellitus (*P* < 0.05).

**Conclusions:**

In this retrospective immunohistochemical analysis of premenopausal women, p16 and PTEN expression in the TAM/TOR group was intermediate between those of EP and EC groups. This pattern may suggest a hyperproliferative polyp phenotype, but remains speculative without confirmatory proliferation biomarkers and molecular validation. Long-term surveillance may be considered for this population, though prospective cohort data are needed to confirm its clinical utility. p16 was positively associated with age, blood lipids, and BMI, and negatively associated with abnormal uterine bleeding and endometrial thickness; PTEN was negatively associated with menopausal status and diabetes mellitus. These preliminary associations provide exploratory clues for endometrial follow-up biomarker research.

## 1 Introduction

Based on GLOBOCAN 2022 statistical data (1), the incidence of breast cancer ranks first among malignant tumors in women. In China, the disease spectrum of breast cancer shows a trend of increasing prevalence in younger populations (2, 3). Surgical treatment remains the first-line option for breast cancer management, while postoperative endocrine therapy serves as a crucial adjuvant treatment for patients with hormone receptor-positive breast cancer (4). Endocrine therapy for breast cancer involves the suppression of endogenous estrogen levels or the blockade of estrogen binding to estrogen receptors on breast cancer cells. It is a systemic antitumor therapy to inhibit the proliferation of hormone receptor-positive breast cancer cells, delay tumor progression, and reduce the risk of recurrence. For patients receiving endocrine therapy, endometrial safety deserves close attention; meanwhile, gynecologists and obstetricians should attach great importance to the reproductive demands of young breast cancer patients.

Based on pharmacological mechanisms, our study primarily investigates selective estrogen receptor modulators (SERMs). SERMs include tamoxifen (TAM) and toremifene (TOR). As an estrogen receptor (ER) antagonist, TAM competes with estradiol for binding to ERα—a key therapeutic target in breast cancer. By inducing conformational changes in ERα, TAM inhibits ER-coactivator interactions, thereby suppressing breast cancer progression and delaying tumor growth. ERβ, an ERα-homologous nuclear receptor expressed in the endometrium, shares structural similarities with ERα and can bind TAM, potentially causing endometrial hyperplasia, endometrial polyps (EP), or endometrial cancer (EC) (5); EP is the most common (6). TOR, with an optimized chloroethyl side chain, exhibits enhanced hydrophobic interactions, improved hydrogen bonding, lower reliance on hepatic metabolism, and stabilized receptor antagonistic conformations. These properties increase ER affinity, conferring stronger anti-estrogenic activity and weaker estrogenic effects, making TOR indicated for postmenopausal patients with ER-positive or ER-unknown metastatic breast cancer. Although TOR theoretically reduces endometrial lesion risk compared to TAM, Hong (7) found that the overall response rate was 87.2% in the TOR group and 80.0% in the TAM group. No statistically significant difference was observed between the two groups (*P* = 0.348). Consistent with Mao (8), TOR exhibits endometrial toxicity comparable to TAM; thus, endometrial monitoring remains necessary during TOR therapy.

The present study aimed to perform immunohistochemical (IHC) analysis of endometrial polyps in breast cancer patients receiving endocrine therapy, explore the expression profiles of p53, PTEN, and p16 and their associations with clinical factors, and provide preliminary evidence to support outpatient follow-up, reproductive function preservation in young breast cancer patients, and early clinical intervention.

## 2 Methods

### 2.1 Study design and participants

This study included 93 breast cancer patients receiving endocrine therapy who received regular follow-up in the Gynecology Outpatient Department of Yangming Hospital Affiliated with Ningbo University from December 1, 2022, to December 31, 2023. All enrolled patients were stratified by medication type and menopausal status into premenopausal and postmenopausal subgroups (TAM and TOR subgroups for each menopausal status). All patients underwent hysteroscopic examination, with postoperative pathological examination and IHC detection of p16, p53, and PTEN. Meanwhile, 60 patients with sporadic EP (control group A) and 48 patients with type I EC (9) (control group B) who received surgical treatment in our hospital were enrolled; each control group was stratified by menopausal status (premenopausal/postmenopausal). Due to the delayed effects of SERMs on the endometrium, the observation group was followed up during treatment and for 1 year after treatment discontinuation.

### 2.2 Inclusion and exclusion criteria

**Figure 1:**
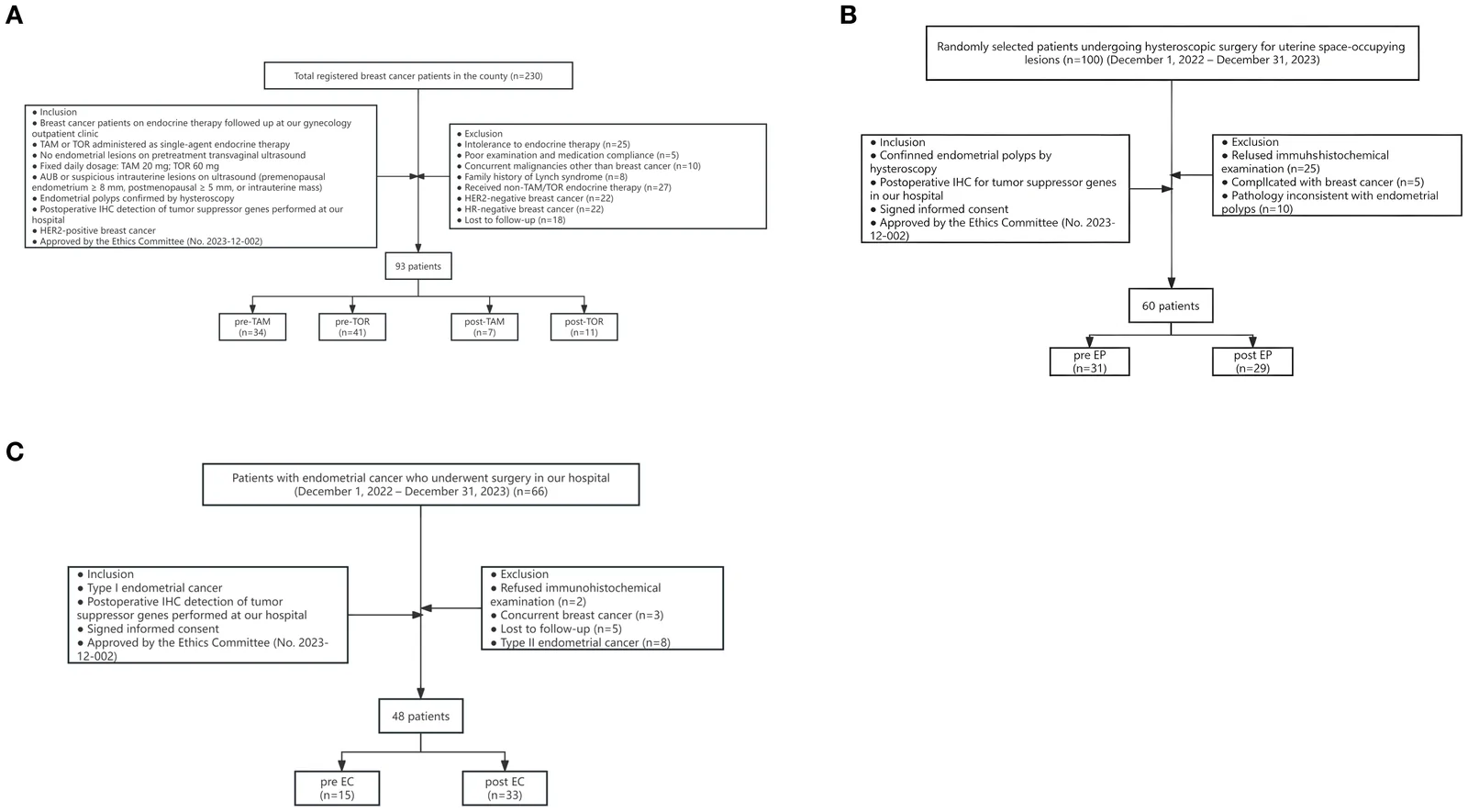
Flowchart of inclusion process of this study. [**(A)** SERM group, **(B)** EP control group, **(C)** EC control group]. TAM, tamoxifen; TOR, toremifene; EP, endometrial polyps; EC, endometrial carcinoma.

Inclusion criteria: 1. Patients receiving endocrine therapy were followed up at our hospital’s Gynecology Outpatient Department. 2. Patients received TAM or TOR as single-agent endocrine therapy. 3. No evidence of endometrial disease confirmed by transvaginal ultrasound prior to treatment initiation. 4. Daily drug dosage: TOR 60 mg; TAM 20 mg. 5. Patients presented with either suspicious intrauterine lesions on ultrasound (premenopausal endometrial thickness ≥ 8 mm, postmenopausal ≥ 5 mm, or intrauterine masses) or abnormal uterine bleeding (AUB). 6. Patients had a confirmed diagnosis of endometrial polyps by hysteroscopy. 7. Patients underwent postoperative IHC detection of p53, PTEN, and p16 at our hospital. 8. Patients had pathologically confirmed human epidermal growth factor receptor 2 (HER2)-positive breast cancer. All the above criteria must be satisfied for patient enrollment. Patients with HER2-negative breast cancer were excluded from the endocrine therapy cohort. This criterion was applied to eliminate confounding factors from heterogeneous HER2 status: the HER2 pathway has well-documented crosstalk with estrogen receptor signaling, which can alter endometrial responses to SERM treatment and interfere with the interpretation of p16/PTEN expression patterns. Restricting enrollment to HER2-positive patients standardized baseline tumor molecular profiles and improved cohort homogeneity for targeted biomarker analysis. Exclusion criteria: 1. Patients intolerant to endocrine therapy. 2. Patients with poor compliance with examinations and medication administration. 3. Patients with concurrent tumors in other sites other than breast cancer. 4. Lynch syndrome family history. 5. Patients who received endocrine therapy other than TAM/TOR. 6. HER2-negative patients. 7. Hormone receptor (HR)-negative patients. 8. Patients lost to follow-up. This study was approved by the Ethics Committee of The Affiliated Yangming Hospital of Ningbo University (No. 2023-12-002); all patients provided written informed consent. Patient screening and enrollment procedures are summarized in **Figure 1**.

### 2.3 Equipment and reagents

#### 2.3.1 Equipment

GE Voluson E8 color Doppler ultrasound diagnostic system, Leica ASP300 S automated tissue processor, HistoStar tissue embedding machine, HistoCore MULTICUT rotary microtome, Dako CoverStainer fully automated slide staining system, BOND-MAX fully automated IHC & ISH staining System, and YD-AB2 slide flotation & drying oven. KARL STORZ 4.0 mm HOPKINS II 30° hysteroscope.

#### 2.3.2 Reagents

PTEN (origin: rabbit monoclonal antibody, brand: Beijing Zhongshan, clone number: D4.3), p53, p16 (origin: murine monoclonal antibody, brand: Fuzhou Maixin, p16 clone number: MX007, p53 clone number: MX008), immunohistochemical chromogenic reagent (origin: United Kingdom, brand: Leica).

### 2.4 Sample collection

Patients were grouped according to the established ranking criteria. All surgical procedures were performed by physicians with the same professional qualification in our department: the observation group and control group A underwent hysteroscopy, while control group B received the standard surgical procedure for endometrial cancer. All tissue specimens were immediately fixed in 4% formalin. Subsequent processing included dehydration, paraffin infiltration, and paraffin embedding, followed by continuous sectioning at a thickness of 3 μm. Hematoxylin-eosin (HE) staining and IHC staining were performed on the sections. The staining results were independently evaluated by two senior pathologists with equivalent experience from our hospital in a double-blind manner.

### 2.5 Staining procedure and quality control

#### 2.5.1 Tissue preparation

Surgically resected specimens were immediately fixed in 10% neutral buffered formalin and promptly delivered to the pathology department. Following gross dissection on a standard pathology workstation, tissue samples were processed on a Leica ASP300 S automated tissue processor with the following protocol: refixation in formalin at 45 °C for 90 min; rinsing with purified water at 37 °C for 10 min; gradient dehydration with 70%, 85% and 95% ethanol at 45 °C (three cycles, lasting 2 h, 2 h and 3 h respectively), followed by two cycles of absolute ethanol dehydration at 45 °C for 1 h per cycle; xylene clearance at 37 °C for two cycles (50 min and 60 min respectively); and paraffin infiltration at 63 °C for three cycles (45 min, 60 min and 105 min respectively). Tissue blocks were embedded in molten paraffin using a HistoStar embedding system, and serially cut into 3 μm-thick sections with a HistoCore MULTICUT rotary microtome.

#### 2.5.2 Hematoxylin and eosin staining

HE staining was performed on a Dako CoverStainer fully automated slide staining system. Slides were baked at 68 °C for 15 min, deparaffinized in two changes of xylene (3 min each), and rehydrated through a graded ethanol series (absolute, 95%, 70%; 2 min each), followed by two rinses with double-distilled water. Slides were stained with hematoxylin for 2.5 min, treated with bluing reagent for 2 min, rinsed in double-distilled water for 5 min, dehydrated in 70% ethanol for 1 min, and counterstained with eosin for 3.5 min. After sequential dehydration in 95% ethanol (30 s) and three changes of absolute ethanol (50 s, 1 min, 1.5 min), slides were cleared in xylene for 3 min, air-dried briefly, and mounted with neutral balsam.

#### 2.5.3 IHC and antigen retrieval

IHC staining was carried out on a BOND-MAX automated IHC stainer. Slides were deparaffinized with BOND Dewax Solution, ethanol and wash buffer at room temperature for 5 min. Antigen retrieval was performed using Epitope Retrieval Solution 2 (ER2) at 100 °C for 20 min, followed by natural cooling to room temperature for 12 min and a 3-min rinse with wash buffer at 35 °C. Endogenous peroxidase activity was blocked with hydrogen peroxide solution at room temperature for 5 min, followed by a 3-min buffer wash at room temperature. For each slide, 100 μl of primary antibody working solution (p53, p16, and PTEN) was applied and incubated at room temperature for 20 min. After four sequential buffer washes (30 s, 1 min, 3 min, 3 min), enhancer was added and incubated for 8 min at room temperature, followed by three 2-min buffer washes. Biotinylated secondary antibody was then applied for 8 min at room temperature, and slides were washed three times with buffer (1 min, 2 min, 2 min). After alternating washes with deionized water and wash buffer, chromogenic detection was performed with 3, 3’-diaminobenzidine (DAB). The reaction was terminated with tap water, followed by hematoxylin counterstaining, gradient dehydration, air-drying and mounting with neutral balsam.

### 2.6 Pathological film reading standard

In IHC staining, p53 showed nuclear positivity, characterized by brownish-yellow granules in the cell nuclei. p16 and PTEN exhibited positive staining localized in both the cytoplasm and nucleus, also presenting as brownish-yellow granules. Interpretation of p53 IHC results: a 3-tier interpretation system is recommended: overexpression and complete absence (confirmed by positive internal controls including lymphocytes, fibroblasts, or endothelial cells) are both regarded as aberrant/mutation-type; both indicate dysfunction of the *TP53* gene. They exhibit similar prognostic significance and are associated with increased tumor aggressiveness. In contrast, expression between these two extremes represents the wild-type pattern, characterized by a mixture of negative, weakly positive, and strongly positive cells. Interpretation of p16, PTEN IHC results: Semi-quantitative scoring was employed for result interpretation. The percentage of positively stained cells relative to the total number of tissue cells was quantified under high-power fields (HPFs). Following a full examination of the entire tissue section, 10 HPFs were randomly selected and the mean value was calculated. A positive staining result was defined as > 10% positive cells, which corresponded to a percentage area score ≥ 1. No digital image-based quantitative analysis was applied in this study. The final score was calculated as the product of two independent parameters: staining intensity and percentage of positive cells. The scoring criteria for each parameter were defined as follows: Staining intensity: Scored as 0 (no staining), 1 (pale yellow), 2 (brownish yellow), 3 (brownish black). Percentage of positive cells: Scored as 1 (≤ 10%), 2 (11%–50%), 3 (51%–75%), 4 (76%–100%). The combined immunohistochemical score ranged from 0 to 12 and was categorized into four expression grades: Grade 0 (negative, −): 0 ≤ combined score < 3; Grade 1 (weak positive, +): 3 ≤ combined score < 6; Grade 2 (moderate positive, ++): 6 ≤ combined score < 8; Grade 3 (strong positive, +++): 8 ≤ combined score ≤ 12. All immunohistochemical sections were blindly reviewed by two experienced pathologists without access to patients’ clinical data. Weighted Cohen’s kappa coefficients were calculated to quantify inter-observer concordance for semi-quantitative IHC scoring of p16, p53, and PTEN. The weighted kappa values reached 0.821 (p16), 0.831 (p53), and 0.824 (PTEN). These results are summarised in **Table 1**.

**Table 1:** Inter-observer agreement of semi-quantitative immunohistochemical scoring assessed by weighted Cohen’s κ test.

Biomarker	Scoring parameter	Weighted κ (95% CI)	SE	Z statistic	*P* value
p16	Percentage of positive cells	0.800 (0.692–0.909)	0.055	5.832	< 0.001
	Staining intensity	0.835 (0.626–1.000)*	0.107	6.001	< 0.001
	Combined semi-quantitative score	0.821 (0.718–0.923)	0.052	5.854	< 0.001
p53	Percentage of positive cells	0.770 (0.637–0.903)	0.068	5.462	< 0.001
	Staining intensity	0.823 (0.708–0.938)	0.059	5.834	< 0.001
	Combined semi-quantitative score	0.831 (0.729–0.932)	0.052	5.890	< 0.001
PTEN	Percentage of positive cells	0.803 (0.686–0.920)	0.060	5.805	< 0.001
	Combined semi-quantitative score	0.824 (0.712–0.935)	0.057	5.920	< 0.001

Inter-rater concordance between two blinded pathologists was quantified using weighted Cohen’s κ coefficients. κ, weighted kappa value; CI, confidence interval; SE, standard error. *The upper bound of the 95% CI was capped at 1.000, the theoretical maximum value of κ. All *P* < 0.001 indicated statistically significant inter-observer consistency.

For quality control of immunohistochemical staining using positive and negative controls, the endogenous negative control strategy was primarily employed. This control comprises normal cellular and tissue components natively present on the same test tissue section, which do not express the target antigen under physiological conditions. This approach obviates the need for separately prepared negative control slides, enables direct verification of intra-section staining specificity, and mitigates intra-batch technical variability. For specimens lacking adequate normal tissue suitable for use as an endogenous control, external control-based quality control was explicitly applied to these samples, and endogenous negative controls were not arbitrarily assigned.

### 2.7 Statistical analysis

All statistical analyses were performed using SPSS 21.0 software, with a two-tailed *P* < 0.05 considered statistically significant. Graphs were generated using R software (version 4.3.3). Data were presented and analyzed as follows: categorical data (baseline characteristics): expressed as n (%), with intergroup comparisons conducted via the chi-square test. Fisher’s exact test was used if the expected frequency in any cell was < 5. Continuous data: normally distributed data: expressed as mean ± standard deviation ( $\bar{x}$ *± s*), with intergroup comparisons using one-way analysis of variance. Non-normally distributed data: expressed as median (M) and quartiles (*P25*, *P75*), with intergroup comparisons via the Kruskal-Wallis H test. PTEN, p16: Semi-quantitative scoring data: The Kruskal-Wallis H rank sum test was used for the overall comparison among multiple groups. Intergroup pairwise comparisons were performed using the Mann-Whitney U non-parametric test. p53: intergroup comparisons conducted via the Fisher’s exact test. Correlation analysis: Spearman’s rank correlation analysis was used to assess correlations between variables.

## 3 Results

Baseline characteristics of the observation group and control group are presented in **Tables 2**, **3**. *P* < 0.05 is considered statistically significant. When stratified by menopausal status, no statistically significant differences in baseline characteristics were identified across the study groups in either the premenopausal or postmenopausal subgroup, indicating favorable intergroup comparability in each stratum.

**Table 2:** Comparison of baseline data among the premenopausal groups.

Group	Age (y)	BMI (kg/m^2^)	Deliveries	Hypertension	Diabetes
	(*± s*)	(*± s*)	M (*P25, P75*)	n (%)	n (%)
TAM (n = 34)	47.59 ± 5.34	22.42 ± 2.82	1 (1, 1)	2 (5.9%)	0 (0.00%)
TOR (n = 41)	47.95 ± 4.80	22.58 ± 2.69	1 (1, 1)	3 (7.3%)	1 (2.44%)
EP (n = 31)	46.1 ± 3.59	23.14 ± 3.52	1 (1, 2)	2 (6.5%)	0 (0.00%)
EC (n = 15)	48.33 ± 6.06	24.75 ± 2.32	1 (1, 1.5)	3 (20%)	1 (6.67%)
*P* value	*P* = 0.423	*P *= 0.060	*P *= 0.308	*P *= 0.367	*P *= 0.312

**Table 3:** Comparison of baseline data among the postmenopausal groups.

Group	Age (y)	BMI (kg/m^2^)	Deliveries	Hypertension	Diabetes
	(*± s*)	(*± s*)	M (*P25, P75*)	n (%)	n (%)
TAM (n = 7)	53.57 ± 9.40	22.84 ± 2.66	1 (1, 1)	2 (28.60%)	1 (14.30%)
TOR (n = 11)	56.18 ± 6.43	25.09 ± 3.61	1 (1, 2)	0 (0.00%)	2 (18.20%)
EP (n = 29)	59.93 ± 6.80	24.59 ± 3.85	2 (1, 2.5)	7 (24.10%)	6 (20.70%)
EC (n = 33)	58.74 ± 6.90	24.23 ± 3.72	2 (1, 2)	13 (39.40%)	4 (12.10%)
*P* value	*P *= 0.074	*P *= 0.620	*P *= 0.094	*P* = 0.083	*P* = 0.829

Comparison of IHC expression of p53: Fisher’s exact test was performed to compare the distribution of p53 expression patterns (wild-type and mutant-type) among the TAM, TOR, EP, and EC groups. The results showed that there was no statistically significant difference among the four groups in both the premenopausal subgroup (*P* = 0.687) and the postmenopausal subgroup (*P* = 0.229). The wild-type p53 expression pattern was predominant in all enrolled patients, with a mutation rate consistent with that of the population reported in previous studies (10). These results are summarised in **Tables 4**, **5**.

**Table 4:** Comparison of p53 staining pattern in each premenopausal group.

Characteristic	TAM group (n=34)	TOR group (n=41)	EP group (n=31)	EC group (n=15)	Fisher’s exact test *P*-value
Wild-type	30 (88.24%)	36 (87.80%)	25 (80.65%)	13 (86.67%)	0.687
Mutant-type	4 (11.76%)	5 (12.20%)	6 (19.35%)	2 (13.33%)	

**Table 5:** Comparison of p53 staining pattern in each postmenopausal group.

Characteristic	TAM group (n=7)	TOR group (n=11)	EP group (n=29)	EC group (n=33)	Fisher’s exact test *P*-value
Wild-type	6 (85.71%)	8 (72.73%)	25 ( 86.21%)	23 (69.70%)	0.229
Mutant-type	1 (14.29%)	3 (27.27%)	4 (13.79 %)	10 (30.30%)	

**Figure 2:**
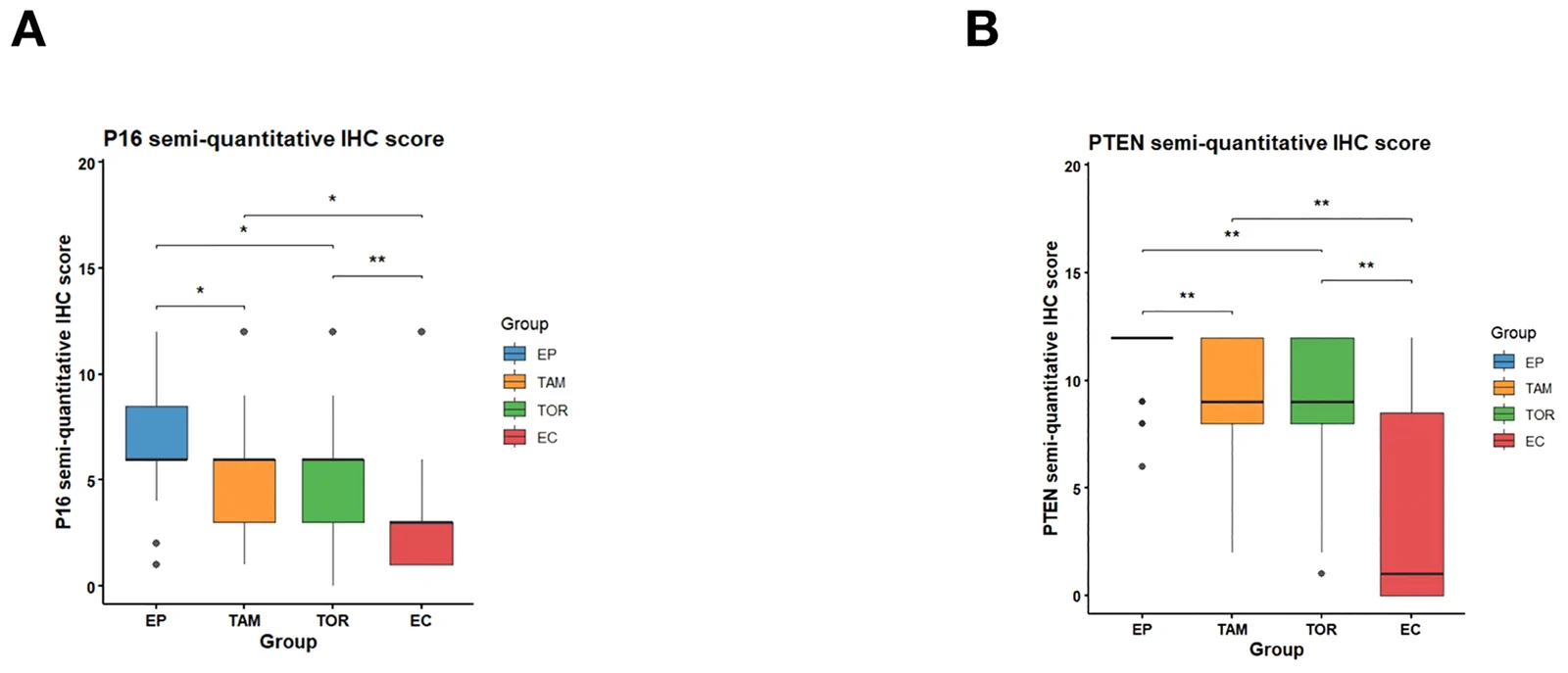
Box plot comparing p16 **(A)** and PTEN **(B)** semi-quantitative IHC score among premenopausal groups.^*^P<0.05, ^**^P<0.01, TAM: tamoxifen, TOR, toremifene; EP, endometrial polyps; EC, endometrial carcinoma.

**Figure 3:**
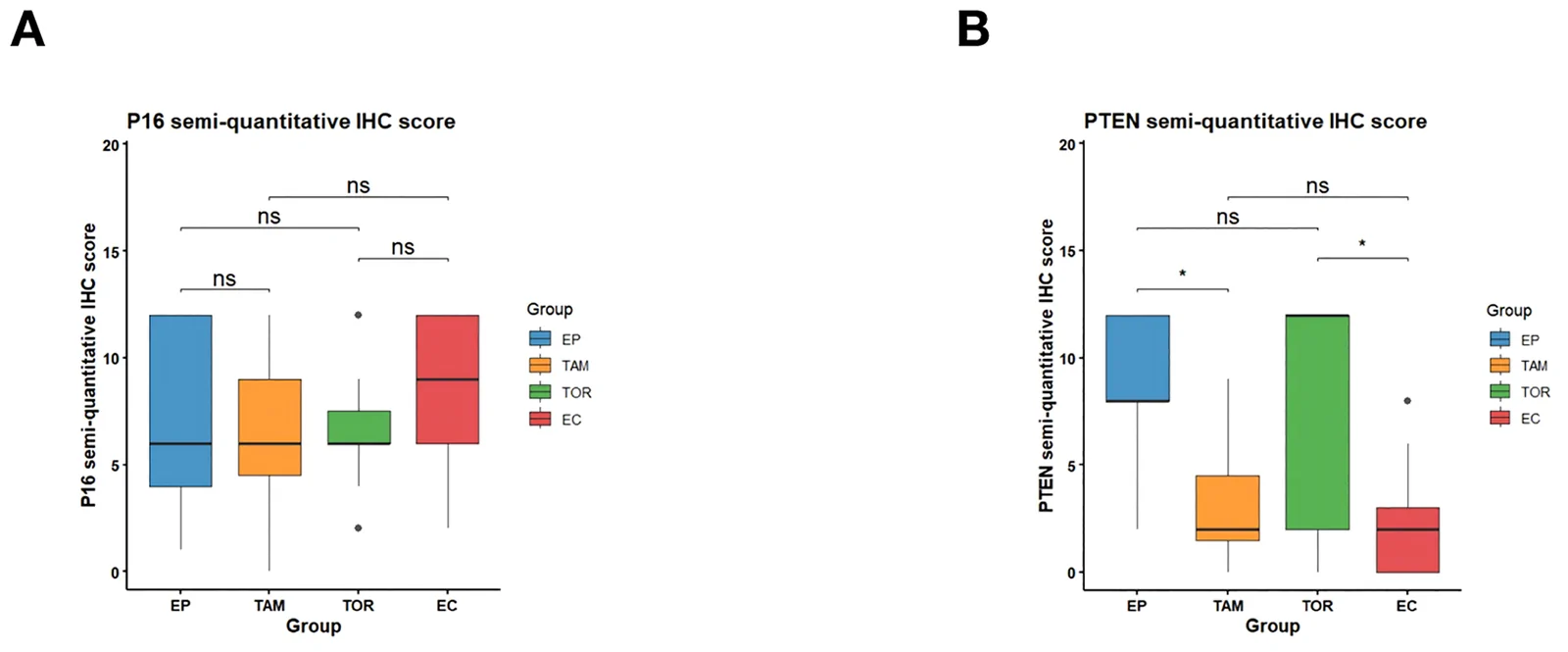
Box plot comparing p16 **(A)** and PTEN **(B)** semi-quantitative IHC score among postmenopausal groups. *P<0.05, ns, not significant; P ≥ 0.05. TAM, tamoxifen; TOR, toremifene; EP, endometrial polyps; EC, endometrial carcinoma.

For the premenopausal subgroup, the Kruskal-Wallis H rank sum test revealed statistically significant differences in the semi-quantitative IHC scores of both p16 and PTEN among the four groups (p16: *H* = 20.654, *df* = 3, *P* < 0.001; PTEN: *H* = 26.396, *df* = 3, *P* < 0.001). Further pairwise comparisons were performed using the Mann-Whitney U test, which showed that semi-quantitative IHC scores of p16 in the TAM and TOR groups were significantly lower than those in the EP group (TAM: Holm-adjusted *P* = 0.038; TOR: Holm-adjusted *P* = 0.032), while significantly higher than those in the EC group (TAM: Holm-adjusted *P* = 0.033; TOR: Holm-adjusted *P* = 0.005). Semi-quantitative IHC scores of PTEN in the TAM and TOR groups were significantly lower than those in the EP group (TAM: Holm-adjusted *P* = 0.004; TOR: Holm-adjusted *P* < 0.006), while significantly higher than those in the EC group (TAM: Holm-adjusted *P* = 0.012; TOR: Holm-adjusted *P =* 0.012). For the postmenopausal subgroup, the Kruskal-Wallis H rank sum test showed no statistically significant difference in p16 expression among the four groups (*H* = 6.792, *df* = 3, *P* = 0.079), while a significant difference was observed in PTEN expression across the groups (*H* = 35.858, *df* = 3, *P* < 0.001). Further pairwise comparisons were performed using the Mann-Whitney U test, which showed that the semi-quantitative IHC score of PTEN in the TAM group was significantly lower than that in the EP group (Holm-adjusted *P* = 0.010); the semi-quantitative IHC score of PTEN was significantly higher in the TOR group than in the EC group (Holm-adjusted *P* = 0.028). These results are summarised in **Tables 6**–**9**. The box plots comparing the semi-quantitative IHC scores of p16 and PTEN between groups are shown in **Figures 2**, **3**. *: *P* < 0.05, the difference is statistically significant, **: *P* < 0.01, the difference is highly statistically significant; ***: *P* < 0.001, the difference is extremely statistically significant, ****: *P* < 0.0001, the difference is very highly statistically significant, ns: *P* ≥ 0.05, the difference is not statistically significant.

**Table 6:** Overall comparison of semi-quantitative IHC scores of p16 and PTEN among the premenopausal groups.

Group	n	p16 semi-quantitative IHC score M*(P25,P75*)	PTEN semi-quantitative IHC score M*(P25,P75*)
TAM	34	6(3,6.75)	9(7.5,12)
TOR	41	6(3,6)	9(7,12)
EP	31	6(6,9)	12(12,12)
EC	15	3(1,3)	1(0,9)
Kruskal-Wallis H	—	*H*=20.654,*df*=3,*P*<0.001	*H*=26.396,*df*=3,*P*<0.001

**Table 7:** Pairwise comparisons between groups of semi-quantitative IHC scores of p16 and PTEN among the premenopausal groups.

Compare	p16			PTEN		
	*Z*	Raw *P* value	Holm Adj.*P*	*Z*	Raw *P* value	Holm Adj.*P*
TAM VS TOR	0.344	0.731	0.731	0.229	0.819	0.819
TAM vs EP	2.349	0.019	0.038	3.280	0.001	0.004
TAM vs EC	2.559	0.011	0.033	2.866	0.004	0.012
TOR vs EP	2.655	0.008	0.032	3.577	<0.001	<0.006
TOR vs EC	3.210	0.001	0.005	2.851	0.004	0.012
EP vs EC	4.037	<0.001	<0.006	4.483	<0.001	<0.006

**Table 8:** Overall comparison of semi-quantitative IHC scores of p16 and PTEN among the postmenopausal groups.

Group	n	p16 semi-quantitative IHC score M*(P25,P75*)	PTEN semi-quantitative IHC score M*(P25,P75*)
TAM	7	6(3,9)	2(1,6)
TOR	11	6(6,9)	12(2,12)
EP	29	6(4,12)	8(7,12)
EC	33	9(6,12)	2(0,3)
Kruskal-Wallis H	—	*H*=6.792,*df*=3,*P*=0.079	*H*=35.858,*df*=3,*P*<0.001

**Table 9:** Pairwise comparisons between groups of semi-quantitative IHC scores of PTEN among the postmenopausal groups.

Compare	PTEN		
	*Z*	Raw *P* value	Holm Adj.*P* value
TAM VS TOR	1.534	0.151	0.453
TAM vs EP	2.999	0.002	0.010
TAM vs EC	0.935	0.382	0.764
TOR vs EP	0.047	0.976	0.976
TOR vs EC	2.697	0.007	0.028
EP vs EC	5.960	<0.001	0.006

**Figure 4:**
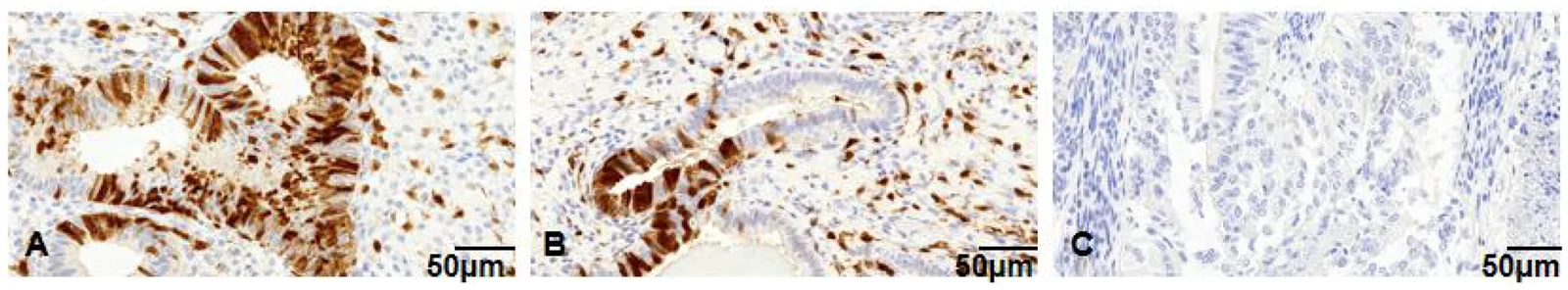
Representative immunohistochemical staining of p16 in premenopausal patients. **(A)** Endometrial polyp group; **(B)** Endocrine therapy group; **(C)** Endometrial carcinoma group. Photomicrographs were obtained at 400×, scale bar = 50 μm. **(A)** represent strong positive; **(B)** represent moderate positive; **(C)** represent negative according to the semi-quantitative scoring system.

**Figure 5:**
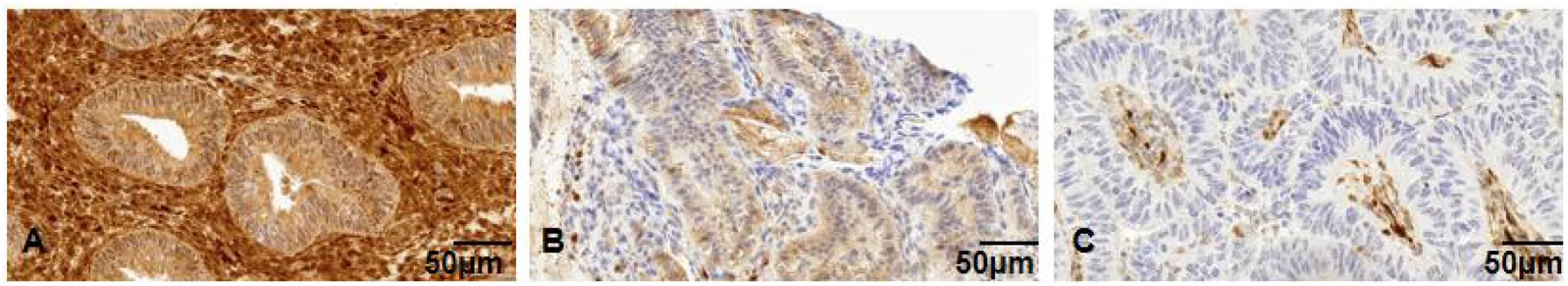
Representative immunohistochemical staining of PTEN in premenopausal patients. **(A)** Endometrial polyp group; **(B)** Endocrine therapy group; **(C)** Endometrial carcinoma group. Photomicrographs were obtained at 400×, scale bar = 50 μm. **(A)** represent strong positive; **(B)** represent moderate positive; **(C)** represent weak positive according to the semi-quantitative scoring system.

Among premenopausal patients, representative immunohistochemical staining of p16 and PTEN are showed in **Figure 4** and **Figure 5**. For p16, strong positive staining was observed in the EP group (**Figure 4A**), and moderate positive immunoreactivity was observed in the SERM group (**Figure 4B**). In the EC group (**Figure 4C**), absent p16 expression was present in a subset of specimens, reflecting heterogeneous p16 status within the EC cohort. For PTEN, strong positive expression was observed in the EP group (**Figure 5A**), moderate positive expression in the SERM group (**Figure 5B**), and weak positive expression in the EC group (**Figure 5C**).

**Figure 6:**
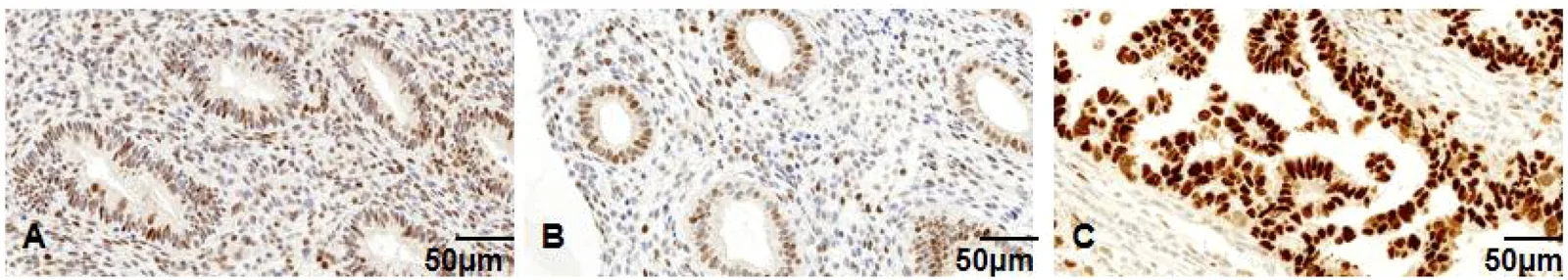
Representative immunohistochemical staining of p53 in premenopausal patients. **(A)** Endometrial polyp group; **(B)** Endocrine therapy group; **(C)** Endometrial carcinoma group. Photomicrographs were obtained at 400×, scale bar = 50 μm. **(A)** and **(B)** represent wild-type pattern; **(C)** represent mutant-type pattern.

The immunohistochemical expression patterns of p53 across groups are shown in **Figure 6**. Samples in **Figures 6A, B** exhibit a typical wild-type p53 staining pattern, whereas the sample in **Figure 6C** demonstrates a mutant-type p53 pattern. Notably, the overall prevalence of p53 alterations was low across all enrolled cases, which accounts for the absence of a statistically significant difference among the three groups.

**Figure 7:**
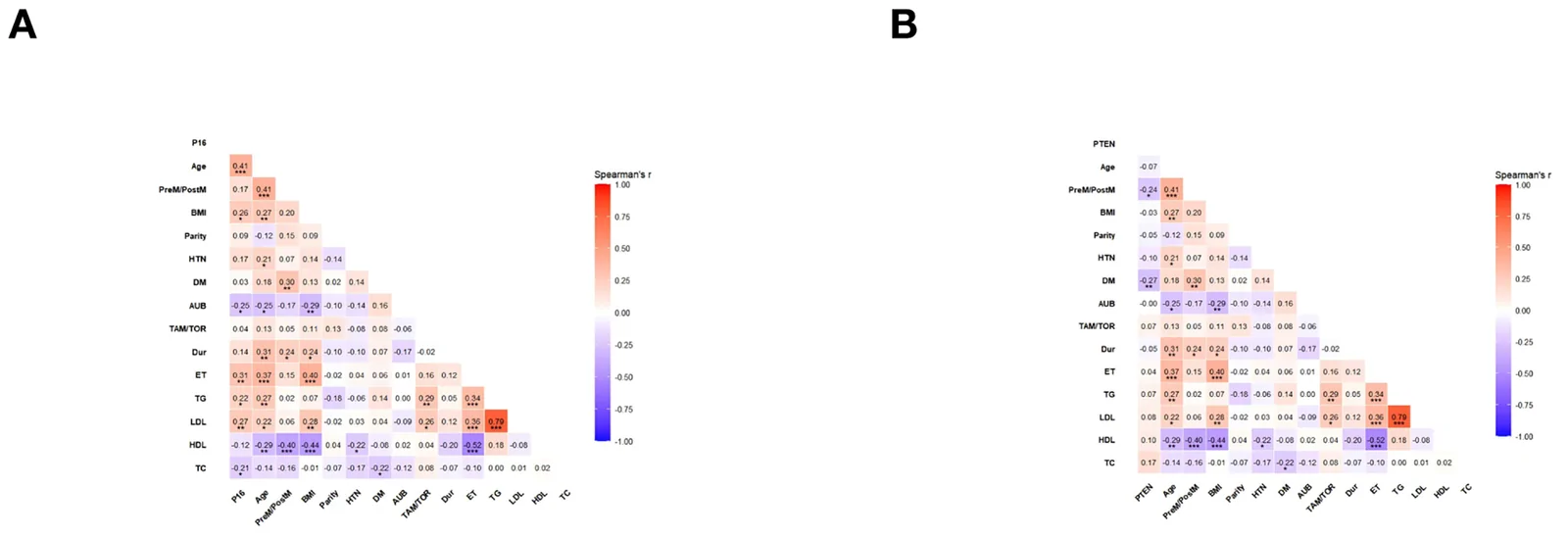
Heatmap of p16 **(A)**, PTEN **(B)** related factors after endocrine therapy for breast cancer. ^*^P<0.05; ^**^P<0.01;^***^P<0.0001; TAM, tamoxifen; TOR, toremifene; EP, endometrial polyps; EC, endometrial carcinoma; Pre M/Post M, Menopausal status; dur, duration; HTN, hypertension; DM, diabetes; AUB, abnormal uterine bleeding; ET, endometrial thickness; TG, triglyceride; LDL, low-density lipoprotein; TC, total cholesterol; HDL, High-density lipoprotein.

Spearman’s rank correlation analysis was performed to investigate the association between the semi-quantitative IHC scores of p16 and PTEN and the clinicopathological characteristics of patients in the observation cohort (endocrine therapy-treated group). For p16 expression, its semi-quantitative IHC score exhibited a positive correlation with age (*r* = 0.406, *P* < 0.001), triglyceride (TG) level (*r* = 0.309, *P* = 0.003), low-density lipoprotein (LDL) level (*r* = 0.275, *P* = 0.008), total cholesterol (TC) level (*r* = 0.218, *P* = 0.036), and BMI (*r* = 0.265, *P* = 0.023). Conversely, p16 expression was negatively correlated with the presence of abnormal uterine bleeding (AUB) (*r* = -0.245, *P* = 0.018) and endometrial thickness (*r* = -0.205, *P* = 0.048). PTEN expression was significantly and negatively correlated with menopausal status (*r* = -0.239, *P* = 0.021) and diabetes mellitus (*r* = -0.267, *P* = 0.010). These results are summarised in **Table 10**. The correlation matrix of p16 and PTEN expression levels with the aforementioned clinicopathological parameters was visualized using a heatmap, as presented in **Figure 7**.

**Table 10:** Correlation analysis of p16, PTEN and clinical characteristics of patients with endometrial polyps after endocrine therapy.

Clinical characteristics	p16		PTEN	
	*r*	*P* value	*r*	*P* value
age( y)	0.406	<0.001	-0.066	0.529
Menopausal status	0.168	0.107	-0.239	0.021
BMI (kg/m^2^)	0.265	0.023	-0.031	0.768
duration	0.144	0.169	-0.048	0.651
parity	0.086	0.414	-0.053	0.613
hypertension	0.170	0.103	-0.100	0.338
diabetes	0.034	0.743	-0.267	0.010
abnormal uterine bleeding	-0.245	0.018	-0.002	0.988
TAM/TOR	0.039	0.709	-0.074	0.483
endometrial thickness (mm)	-0.205	0.048	0.171	0.101
TG (mmol/L)	0.309	0.003	0.043	0.681
LDL (mmol/L)	0.275	0.008	0.084	0.424
HDL (mmol/L)	-0.125	0.234	0.098	0.351
TC (mmol/L)	0.218	0.036	0.066	0.527
longest diameter (mm)	0.203	0.052	0.068	0.521

## 4 Discussion

### 4.1 Comparison of p53 IHC expression patterns among three types of endometrial lesions

The primary explanation for the lack of statistically significant differences in p53 expression patterns among the three groups is the inherently low incidence of *TP53* gene alterations across all enrolled estrogen-related endometrial lesions. p53, encoded by the *TP53* tumor suppressor gene, is a core regulator of cell cycle arrest, DNA damage repair, and apoptosis. Aberrant p53 IHC staining patterns, including diffuse strong positivity, complete null staining, and aberrant cytoplasmic staining, are widely recognized as reliable surrogate markers for inactivating or missense mutations of the *TP53* gene (11). Low-grade type I endometrioid endometrial carcinoma, the most common histological subtype of endometrial malignancy, is predominantly estrogen-driven. It is characterized by frequent molecular alterations in *PTEN*, *PIK3CA*, and *KRAS*, whereas *TP53* mutations are rare and largely restricted to high-grade, advanced-stage or recurrent disease (12). Meanwhile, both common EP and breast cancer endocrine therapy-related EP are benign hyperplastic endometrial lesions, in which *TP53* mutations are exceedingly rare (13). Given the uniformly low baseline rate of aberrant p53 expression across the three groups, combined with the inherent limitation of sample size in the present study, no statistically significant between-group differences were detected in our cohort.

### 4.2 Comparison of p16 semi-quantitative IHC score among three types of endometrial lesions

In the premenopausal subgroup, we detected a statistically significant difference in p16 expression. Specifically, *post hoc* pairwise analyses with the Mann-Whitney U test demonstrated that the semi-quantitative IHC score of p16 was significantly lower in the TAM and TOR treatment groups than in the benign EP group, while it was significantly higher in the TAM and TOR cohorts than in the EC group. This heterogeneous expression pattern may reflect differences in endometrial proliferation, genomic instability, and aberrant cell cycle regulation under varying hormonal and therapeutic conditions. In premenopausal women, the endometrium is subject to strong cyclic regulation by estrogen and progesterone. EP is typically characterized by persistent estrogen-driven epithelial proliferation, often accompanied by mild inactivation of cyclin-dependent kinases and compensatory upregulation of p16 expression (14). In contrast, EC, particularly low-grade type I endometrial carcinoma, frequently harbors alterations in cell cycle regulatory genes such as *PTEN*, *PIK3CA*, and *KRAS*. These genetic alterations can disrupt physiological cell cycle checkpoints, thereby leading to downregulated p16 expression (15).

The semi-quantitative IHC scores of p16 in the TAM group and the TOR group were between those in the EP group and the EC group. TAM and TOR can directly or indirectly modulate the endometrial microenvironment in premenopausal patients, eliciting mild-to-moderate proliferation or cellular stress responses (16). Although such alterations are inadequate to induce malignant transformation, they surpass the relatively quiescent status of paracancerous tissues, indicating that moderate p16 expression may reflect borderline proliferation rather than a frank malignant phenotype (17, 18).

In the postmenopausal subgroup, no statistically significant difference was observed in p16 expression. This finding may be attributed to the low intrinsic proliferative activity, weak hormonal stimulation, and slow cell cycle turnover of the postmenopausal endometrium (19). TAM and TOR exert only weak effects on endometrial proliferation and cell cycle regulation, resulting in low p16 expression with no statistically significant difference across all groups. The sample size of postmenopausal subgroups is relatively limited. The reduced sample size weakens the statistical power of intergroup comparisons and may increase the risk of Type II errors and spurious associations. Accordingly, this may be another reason why the postmenopausal subgroup shows no statistical significance, and further validation in large-scale multicenter cohorts is warranted.

### 4.3 Comparison of PTEN semi-quantitative IHC score among three types of endometrial lesions

The findings of this study revealed that in the premenopausal subgroup, the semi-quantitative IHC scores of PTEN in SERM-associated endometrial polyps exhibited an intermediate level between those in the EP group and the EC group, a feature worthy of further investigation for its biological and clinical relevance. The mechanism underlying this difference is as follows: the pathogenesis of EP is mostly associated with focal physiological proliferation driven by high local expression of estrogen receptors (20). Without sustained exogenous pharmaceutical hormonal stimulation, proliferative stress is low, and thus normal PTEN expression is maintained in the vast majority of lesions (21). In premenopausal women, cyclic endogenous estrogen secretion, combined with the partial estrogen agonist activity of TAM/TOR in the endometrium, creates a hormonal microenvironment that sustains endometrial epithelial cells in a chronically proliferative state and increases proliferative stress. According to prior mechanistic research (22), this proliferative milieu may trigger compensatory downregulation of the tumor suppressor gene *PTEN*. Combined with our IHC expression findings, this pathway may serve as a potential mechanistic explanation, rather than a confirmed molecular basis, for the larger volume, higher glandular density, and higher rate of concurrent atypical hyperplasia of SERM-associated polyps observed in clinical practice (23).

However, although the semi-quantitative IHC score of PTEN in SERM-associated polyps was significantly downregulated compared with that in the EP group, it was still markedly higher than that in the EC group. This finding indicates that such lesions only present mild proliferation-related molecular abnormalities, without the loss of PTEN expression or complete functional inactivation characteristic of EC. Therefore, they still fall into the category of benign lesions and have not reached the molecular threshold for malignant transformation. These immunohistochemical expression pattern observations do not constitute functional laboratory data and only deliver preliminary supportive observational clues, rather than direct experimental evidence, to inform clinical strategies that aim to avoid overtreatment of these endometrial lesions. While regular surveillance may be considered for this subgroup, our retrospective dataset lacks long-term progression data and cannot validate that such monitoring effectively prevents malignant transformation.

In addition, reduced PTEN expression can be modulated by multiple confounding factors including chronic inflammation and hormonal stimulation (24). The present study only assessed PTEN expression at the protein level via immunohistochemistry, without further exploration at the genetic level. Further in-depth mechanistic investigations at the gene level are warranted in subsequent research.

Different from the premenopausal subgroup, in the postmenopausal subgroup of this study, only the semi-quantitative IHC score of PTEN in the TOR group was significantly higher than that in the EC group; the semi-quantitative IHC score of PTEN in the TAM group was significantly lower than that in the EP group, with no statistically significant differences observed in all other pairwise comparisons between groups. This phenomenon is related to the inherent physiological characteristics of the postmenopausal endometrium. After menopause, women experience ovarian function failure, with endogenous estrogen levels dropping to an extremely low level, and the endometrium enters a state of physiological atrophy. It inherently has extremely low proliferative activity, significantly diminished responsiveness to hormonal stimulation, and slow cell cycle turnover (19). Against this physiological background, neither common sporadic polyps nor TAM/TOR-associated polyps have sufficient hormonal stimulation to drive abnormal cell proliferation. Accordingly, PTEN expression in both types of polyps is maintained at a relatively stable level without statistically significant differences, and their molecular phenotypes are highly similar. Both are essentially distinct from the phenotype of low/absent PTEN expression that is characteristic of type I endometrial carcinoma (20). Our study found that the semi-quantitative IHC scores of PTEN were significantly lower in the TAM group than in the EP group, and the TOR group showed significantly higher PTEN scores compared with the EC group. No significant difference was observed between the TAM and TOR groups. This pattern may be interpreted as follows. Both TAM and TOR belong to SERMs and can induce endometrial polyp formation, leading to partial overlap in PTEN expression profiles between drug-associated polyps. Although TAM exerts estrogenic agonistic effects on the postmenopausal endometrium and tends to trigger PTEN downregulation, the magnitude of the difference between TAM-related and TOR-related polyps may not be sufficient to reach statistical significance under the current sample size. Compared with spontaneous endometrial polyps (EP), sustained stimulation from TAM significantly disrupts PTEN homeostasis. By contrast, the weaker endometrial agonistic activity of TOR helps preserve PTEN expression to a certain extent. Such pharmacological discrepancies are likely linked to the unique chloroethyl side chain structure of TOR, which stabilizes the antagonistic conformation of estrogen receptors and alleviates undesired estrogen-like actions on the endometrium.

### 4.4 The semi-quantitative IHC score of p16 is associated with age, BMI, blood lipid profiles, AUB, and endometrial thickness in the observation group

p16, a well-characterized cyclin-dependent kinase inhibitor and canonical biomarker of cellular senescence, exerts its tumor suppressor function by arresting cell cycle progression at the G1/S phase, thereby inhibiting uncontrolled cell proliferation (24). In the present study, the semi-quantitative IHC score of p16 was positively correlated with age in endometrial polyps derived from breast cancer patients receiving endocrine therapy. This finding is consistent with the well-defined role of p16 in mediating aging-associated cellular senescence. With advancing age, the accumulation of DNA damage and replicative senescence in endometrial epithelial cells drives constitutive upregulation of p16 expression (17). Moreover, increasing age contributes to shifts in circulating sex hormone concentrations (25) and modified estrogen receptor signaling (26). These biological changes are further shaped by prolonged SERM treatment, which induces weak estrogenic actions within the endometrium (5). Such a unique endocrine milieu may potentiate senescence-related signaling cascades, potentially explaining the positive association between age and p16 expression identified in our cohort.

Notably, we identified a significant positive correlation between the semi-quantitative IHC score of p16 and circulating lipid profiles, including total cholesterol (TC), triglyceride (TG), and low-density lipoprotein (LDL) levels. Endocrine therapy may further perturb systemic lipid metabolism (27). Emerging evidence links dyslipidemia to cellular senescence: oxidized LDL can induce DNA damage and endoplasmic reticulum stress via activation of the *p38 MAPK* pathway, which in turn upregulates p16 expression and triggers irreversible cell cycle arrest (28). This is consistent with the positive correlation observed between lipid levels and p16 expression in this study.

Conversely, p16 expression was negatively correlated with endometrial thickness and the presence of AUB, two well-recognized surrogate markers of endometrial proliferative risk in patients receiving TAM therapy (29). Consistent with prior functional studies, p16 loss or downregulation leads to abrogation of cell cycle control and drives unrestrained endometrial epithelial proliferation, a phenotype that may correspond to thicker endometrium on ultrasound and abnormal uterine bleeding clinically. The inverse associations between p16 expression and these clinical features observed in our univariate correlation analyses only identify p16 as a tentative candidate expression marker, rather than a validated protective biomarker, for stratifying patients at low risk of endocrine therapy-associated endometrial abnormalities. Definitive confirmation of its biomarker utility requires independent prospective validation.

### 4.5 The semi-quantitative IHC score of PTEN is specifically correlated with menopausal status, diabetes mellitus status in the endocrine-treated cohort

In the present study, we identified a negative correlation between the semi-quantitative IHC score of PTEN and both menopausal status and diabetes mellitus in endocrine therapy-associated endometrial polyps.

As a core tumor suppressor negatively regulating the *PI3K/AKT* proliferative pathway, PTEN loss of function is well-established as an early driver event in endometrial tumorigenesis (30). In premenopausal women with relatively high levels of endogenous estrogen, SERMs predominantly exert estrogen-antagonistic actions on endometrial epithelial cells, resulting in mild proliferative stimulation. Upon menopause, circulating endogenous estrogen drops sharply, whereby the weak estrogenic agonistic effect of SERMs serves as the major stimulus for endometrial tissue. Chronic long-standing proliferative stress may lead to PTEN downregulation through epigenetic silencing and enhanced protein degradation (30, 31). Type 2 diabetes mellitus is characterized by insulin resistance and compensatory hyperinsulinemia. Insulin induces persistent overactivation of the *PI3K/AKT* signalling pathway. Sustained pathway activation initiates negative feedback loops that further inhibit PTEN expression and attenuate its capacity to restrain epithelial proliferation (32). Menopause-related hormonal changes and diabetes-associated metabolic disorders may act synergistically to lower PTEN expression and raise the risk of aberrant proliferation of endometrial polyps. Given the cross-sectional design of this study, only correlative associations can be identified, and causality cannot be established. Prospective cohort investigations and fundamental experimental research are required to verify these findings.

### 4.6 Clinical implications of immunohistochemical expression for endometrial risk stratification in endocrine-treated breast cancer patients

Our findings have important clinical implications for the personalized management of breast cancer patients receiving adjuvant endocrine therapy. Currently, endometrial surveillance for this population relies mainly on transvaginal ultrasound measurement of endometrial thickness and AUB, which have limited specificity for detecting premalignant or malignant lesions. The significant correlation between p16 expression and both established endometrial cancer risk factors (age, BMI, metabolic dysregulation) and surrogate markers of endometrial proliferation (endometrial thickness, AUB) suggests that p16 may serve as a complementary molecular biomarker to refine risk stratification. Specifically, patients with low p16 expression may represent a high-risk subgroup that requires more intensive endometrial surveillance, while those with high p16 expression may be candidates for less frequent monitoring, thereby optimizing the allocation of healthcare resources. In addition, the specific correlations between PTEN expression and both menopausal status and diabetes mellitus status highlight the value of incorporating these factors into endometrial risk assessment for patients receiving endocrine therapy. Combining readily available clinical indicators (menopausal status and diabetes) with PTEN immunohistochemical results may help identify high-risk subgroups. Postmenopausal patients with diabetes and low PTEN expression in polyps may require intensified endometrial surveillance, while low-risk individuals can avoid redundant examinations.

## 5 Conclusions

This study examined the immunohistochemical expression of p53, p16, and PTEN in endocrine therapy-associated endometrial polyps. Preliminary data suggest possible involvement of these three biomarkers in the development and progression of these lesions. Our results may deepen the understanding of their immunohistochemical expression and provide tentative clues for the clinical assessment and follow-up of patients on endocrine therapy.

However, several limitations of this study should be acknowledged. First, this is a single-center retrospective study with a relatively limited sample size. In accordance with the widely accepted 10–15 events per variable (EPV) rule, the current sample size is insufficient to support multivariable regression analysis adjusted for confounding factors. Accordingly, only univariate Spearman rank correlation analysis was conducted, and the correlation findings remain exploratory and require further validation in larger cohort studies. Second, only protein expression levels were detected via immunohistochemistry, without in-depth elucidation of the underlying molecular mechanisms. Third, long-term follow-up data are lacking, which to some extent restricts the generalizability of the conclusions and the depth of the study. Fourth, our study has inherent selection bias. All enrolled patients in the endocrine therapy cohort had clinical indications for endometrial biopsy (suspicious ultrasound findings or abnormal uterine bleeding); asymptomatic breast cancer patients receiving endocrine therapy were not included. Therefore, our findings are only generalizable to symptomatic patients receiving SERM treatment, and cannot represent the full spectrum of breast cancer patients undergoing endocrine therapy.

Only univariate Spearman correlation analysis was performed in this study, with no adjustment for potential confounders including age, menopausal status, BMI, lipid profile, parity, and endocrine treatment type. The observed associations between p16/PTEN expression and clinical parameters are preliminary bivariate correlations, which cannot confirm independent predictive effects. Therefore, p16 and PTEN cannot currently be regarded as clinically actionable biomarkers for endometrial follow-up in this population. Further studies with multivariate adjustment and larger sample sizes are required to verify the independent correlation and clinical utility of these indicators.

Future large-scale multicenter prospective studies integrating long-term follow-up and molecular mechanism exploration are warranted to verify the clinical application value of p53, p16, and PTEN, optimize the combined detection strategy for these biomarkers, and develop individualized surveillance and intervention regimens, ultimately improving the diagnosis, treatment and prognosis of patients with endocrine therapy-associated endometrial lesions.

## Data Availability

The raw data supporting the conclusions of this article will be made available by the authors, without undue reservation.

## References

[1] Filho AM Laversanne M Ferlay J Colombet M Piñeros M Znaor A . The GLOBOCAN 2022 cancer estimates: data sources, methods, and a snapshot of the cancer burden worldwide. Int J Cancer. (2025) 156:1336–46. doi: 10.1002/ijc.35278 39688499

[2] Yang Y Liu J Peng M Su F Xie X Liu Z . Introduction of a multicenter online database for non-metastatic breast cancer in China. Sci China Life Sci. (2020) 63:1417–20. doi: 10.1007/s11427-019-1625-1 32088826

[3] Guo R Si J Xue J Su Y Mo M Yang B . Changing patterns and survival improvements of young breast cancer in China and SEER database, 1999-2017. Chin J Cancer Res. (2019) 31:653–62. doi: 10.21147/j.issn.1000-9604.2019.04.09 PMC6736653 31564808

[4] Health Commission of the People's Republic of China . National guidelines for diagnosis and treatment of breast cancer 2022 in China (English version). Chin J Cancer Res (2022) 34(3):151–75. doi: 10.21147/j.issn.1000-9604.2022.03.02 PMC9273574 35873887

[5] Ryu KJ Kim MS Lee JY Nam S Jeong HG Kim T . Risk of endometrial polyps, hyperplasia, carcinoma, and uterine cancer after tamoxifen treatment in premenopausal women with breast cancer. JAMA Netw Open. (2022) 5:e2243951. doi: 10.1001/jamanetworkopen.2022.43951 36441547 PMC9706361

[6] Schwartz LB Krey L Demopoulos R Goldstein SR Nachtigall LE Mittal K . Alterations in steroid hormone receptors in the tamoxifen-treated endometrium. Am J Obstet Gynecol. (1997) 176:129–37. doi: 10.1016/s0002-9378(97)80025-7 9024103

[7] Hong J Huang J Shen L Zhu S Gao W Wu J . A prospective, randomized study of toremifene vs. tamoxifen for the treatment of premenopausal breast cancer: safety and genital symptom analysis. BMC Cancer. (2020) 20:663. doi: 10.1186/s12885-020-07156-x 32677982 PMC7364473

[8] Mao C Yang ZY He BF Liu S Zhou JH Luo RC . Toremifene versus tamoxifen for advanced breast cancer. Cochrane Database Syst Rev. (2012) 7:CD008926. doi: 10.1002/14651858.CD008926.pub2 22786516 PMC8407374

[9] Bokhman JV . Two pathogenetic types of endometrial carcinoma. Gynecol Oncol. (1983) 15:10–7. doi: 10.1016/0090-8258(83)90111-7 6822361

[10] Miranda SP Traiman P Cândido EB Lages EL Freitas GF Lamaita RM . Expression of p53, Ki-67, and CD31 proteins in endometrial polyps of postmenopausal women treated with tamoxifen. Int J Gynecol Cancer. (2010) 20:1525–30. doi: 10.1111/IGC.0b013e3181f7b33b 21119367

[11] Raffone A Travaglino A Cerbone M De Luca C Russo D Di Maio A . Diagnostic accuracy of p53 immunohistochemistry as surrogate of TP53 sequencing in endometrial cancer. Pathol Res Pract. (2020) 216:153025. doi: 10.1016/j.prp.2020.153025 32703491

[12] Cancer Genome Atlas Research Network Kandoth C Schultz N Cherniack AD Akbani R Liu Y . Integrated genomic characterization of endometrial carcinoma. Nature. (2013) 497:67–73. doi: 10.1038/nature12113 23636398 PMC3704730

[13] Kurman RJ Carcangiu ML Herrington CS Young RH . WHO classification of tumours of female reproductive organs, 4th ed. In: WHO Classification of Tumours of Female Reproductive Organs, 4th Ed. International Agency for Research on Cancer (IARC) Press, Lyon (2014).

[14] Stewart CJR Bharat C Crook M . p16 immunoreactivity in endometrial stromal cells: stromal p16 expression characterises but is not specific for endometrial polyps. Pathology. (2015) 47:112–7. doi: 10.1097/PAT.0000000000000211 25551298

[15] Bostan IS Mihaila M Roman V Radu N Neagu MT Bostan M . Landscape of endometrial cancer: molecular mechanisms, biomarkers, and target therapy. Cancers (Basel). (2024) 16:2027. doi: 10.3390/cancers16112027 38893147 PMC11171255

[16] Nijkang NP Anderson L Markham R Manconi F . Endometrial polyps: pathogenesis, sequelae and treatment. SAGE Open Med. (2019) 7:2050312119848247. doi: 10.1177/2050312119848247 31105939 PMC6501471

[17] Safwan-Zaiter H Wagner N Wagner KD . p16INK4A—more than a senescence marker. Life. (2022) 12:1332. doi: 10.3390/life12091332 36143369 PMC9501954

[18] Pleasant V Pearlman MD . Does tamoxifen use increase the risk of endometrial cancer in premenopausal patients? OBG Manag. (2023) 35:17. doi: 10.12788/obgm.0297 38919701 PMC11197877

[19] Yan ZY Zhou WJ Ye JF Xie F Zhang CX Li MQ . Cellular senescence in endometrium: a pivotal regulator in physiological remodeling and pathological disorders. Int J Biol Sci. (2025) 21:6745–58. doi: 10.7150/ijbs.123036 41281750 PMC12631108

[20] Pamidimukkala S Srismitha S Priyadarshini RSG . Immunohistochemical evaluation of phosphatase and tensin homolog (PTEN) expression in endometrial lesions: a cross-sectional study in a tertiary care center in south India. Cureus. (2025) 17:e94512. doi: 10.7759/cureus.94512 41246751 PMC12612564

[21] Yan C Xing C Wei T Zhou H Wang H Liu T . Impact of estrogen and progesterone receptor expression on the incidence of endometrial polyps. Biomarkers Med. (2023) 17:881–7. doi: 10.2217/bmm-2023-0411 38230984

[22] Kübler K Nardone A Anand S Gurevich D Gao J Droog M . Tamoxifen induces PI3K activation in uterine cancer. Nat Genet. (2025) 57:2192–202. doi: 10.1038/s41588-025-02308-w 40846762 PMC12425819

[23] Shen DH . Current research status on pathological diagnosis and malignant transformation for endometrial polyps. Chin J Obstet Gynecol Pediatr (Electron Ed). (2025) 21(4):375–9. doi: 10.3877/cma.j.issn.1673-5250.2025.04.001

[24] Hu Z Tang M Huang Y Cai B Sun X Chen G . SIRT7 facilitates endometrial cancer progression by regulating PTEN stability in an estrogen-dependent manner. Nat Commun. (2025) 16:2989. doi: 10.1038/s41467-025-58317-0 40148340 PMC11950185

[25] Loid M Obukhova D Kask K Apostolov A Meltsov A Tserpelis D . Aging promotes accumulation of senescent and multiciliated cells in human endometrial epithelium. Hum Reprod Open. (2024) 2024:hoae048. doi: 10.1093/hropen/hoae048 39185250 PMC11344589

[26] Motlani V Motlani G Pamnani S Sahu A Acharya N . Endocrine changes in postmenopausal women: a comprehensive view. Cureus. (2023) 15:e51287. doi: 10.7759/cureus.51287 38288203 PMC10823308

[27] Camon C Garratt M Correa SM . Exploring the effects of estrogen deficiency and aging on organismal homeostasis during menopause. Nat Aging. (2024) 4:1731–44. doi: 10.1038/s43587-024-00767-0 39672893 PMC11785355

[28] Moussaoui W Lahmamssi FZ Aynaou H Salhi H El Ouahabi H . Tamoxifen- and triptorelin-induced major hypertriglyceridemia: a case report. Cureus. (2024) 16:e60434. doi: 10.7759/cureus.60434 38469007 PMC10927164

[29] Luo G Xiang L Xiao L . Quercetin alleviates atherosclerosis by suppressing oxidized LDL-induced senescence in plaque macrophage via inhibiting the p38 MAPK/p16 pathway. J Nutr Biochem. (2023) 116:109314. doi: 10.1016/j.jnutbio.2023.109314 36924853

[30] Ali IU . Gatekeeper for endometrium: the PTEN tumor suppressor gene. J Natl Cancer Inst. (2000) 92:861–3. doi: 10.1093/jnci/92.11.861 10841815

[31] Kovalenko TF Morozova KV Ozolinya LA Lapina IA Patrushev LI . The PTENP1 pseudogene, unlike the PTEN gene, is methylated in normal endometrium, as well as in endometrial hyperplasias and carcinomas in middle-aged and elderly females. Acta Naturae. (2018) 10(1):43–50. 29713518 PMC5916733

[32] Kyo S Nakayama K . Endometrial cancer as a metabolic disease with dysregulated PI3K signaling: shedding light on novel therapeutic strategies. Int J Mol Sci. (2020) 21(17):6073. 32842547 10.3390/ijms21176073 PMC7504460

